# ﻿Morpho-molecular analysis of two new species *Deconica* and *Entocybe* in Agaricales from Mount Tianmu, China

**DOI:** 10.3897/mycokeys.109.131298

**Published:** 2024-10-22

**Authors:** Yu-Yu Shen, Zi-Wen Zhang, Wen-Qian Li, Xing-Ning Liu, Fei-Ying Tian, Chun-Mei Pang, Wen-Hong Dai, Yao-Bin Song, Ming Dong

**Affiliations:** 1 Key Laboratory of Hangzhou City for Ecosystem Protection and Restoration, College of Life and Environmental Sciences, Hangzhou Normal University, Hangzhou 311121, China Hangzhou Normal University Hangzhou China; 2 Management Bureau of Mount Tianmu National Nature Reserve, Hangzhou 311311, China Management Bureau of Mount Tianmu National Nature Reserve Hangzhou China; 3 Ecological Security and Protection Key Laboratory of Sichuan Province, Mianyang Normal University, Mianyang 621000, China Mianyang Normal University Mianyang China

**Keywords:** Entolomataceae, Strophariaceae, taxonomy

## Abstract

Two new species of Agaricales, *Deconicaflavum* and *Entocyberoseoalbus*, are described from Mount Tianmu, Zhejiang Province, Eastern China. Two new species are distinct and monophyletic based on morphology and phylogenetic analyses. *Deconicaflavum* differs from other *Deconica* species in that the pileus is brow shallow in the center and stipe with yellowish white fibrils, scatted on litter under coniferous and broad-leaved mixed forest at 1162 m. *Entocyberoseoalbus* is distinguished from other species of *Entocybe* by nearly blue pileus and pinkish-white stipe, scatted on humus in similar forest conditions at 1025 m. The differences are discussed between the two new taxa and their similar species morphologically, and related species phylogenetically.

## ﻿Introduction

Agaricales is the largest order in the kingdom Fungi with nearly 20,000 species (Roskov et al. 2019). The members of the order play essential roles in the ecosystem as saprotrophs, ectomycorrhizae, lichens, and crops cultivated by termites and ants (Kalichman and Matheny 2020). Due to its great diversity, the order has been intensively studied worldwide (Kalichman and Matheny 2020).

The genus *Deconica* (W.G. Sm.) P. Karst. has been placed in the family Strophariaceae of Agaricales (Matheny et al. 2006; Ramírez-Cruz et al. 2013, 2020a) and was initially described as a subgenus of *Agaricus* (Smith 1870). It was subsequently raised to the genus level by Karsten (1879). But for the last several years species of *Deconica* were placed in the genus *Psilocybe* (Fr.) P. Kumm. because of the similarity in their morphology (Moncalvo et al. 2002; Noordeloos 2009). However, molecular studies have shown that the genus *Deconica* is phylogenetically distant from *Psilocybe* (Ramírez-Cruz et al. 2013). The genus *Deconica* is distinguished based on the absence of hallucinogenic compounds (Ramírez-Cruz et al. 2020b). The members of the genus *Deconica* have mycenoid, collybioid, crepidotoid, or omphaloid basidiomata occurring in/on the soil, grasses, mosses, rotten wood, trunks, and dung (Noordeloos 2009; Ramírez-Cruz et al. 2020b). Noordeloos (2011) mentioned that the genus *Deconica* has been divided into three sections namely, *Deconica*, *Melanotus*, and *Merdariae* based on characteristics of basidiomata, basidiospores, pileipellis, and cystidia. However, chrysocystidia have not been mentioned in most sections, except *Deconica* section (Singer 1986). Guzmán (2005) estimated around 133 species in the genus *Deconica* exist worldwide. However, He et al. (2019) and Kalichman and Matheny (2020) reported 44 and 45 taxa worldwide, respectively. Furthermore, *Deconica* was one of the largest genera with an unsequenced generic type (Kalichman and Matheny 2020).

*Entocybe* T. J. Baroni, Hofstetter & Largent is the genus placed in the family Entolomataceae within Agaricales (Baroni et al. 2011). Species of Entocybe were previously placed in section Turfosa, subgenus Entoloma of the genus *Entoloma* (Noordeloos 1992). The genus *Entocybe* was erected based on the morphological and molecular phylogeny-based data (Baroni et al. 2011). The basidiomata of *Entocybe* show slender tricholomatoid or mycenoid to collybioid habit and possess a relatively fragile, appressed fibrillose stipe (Baroni et al. 2011). The basidiospores of *Entocybe* are 6–10 angled in polar view with undulate-pustulate or rounded pustulate surface ornamentation, or ornamentation being composed of broken interconnected ridges with isolated pustules interspersed, which are similar to *Rhodocybe* (Baroni et al. 2011). Additionally, clamp connections are found on the hyphae in all tissues (Baroni and Lamoureux 2013). According to Index Fungorum (http://www.indexfungorum.org), *Entocybe* currently comprises around ten species.

In the past decade, new species, combinations, and records of *Deconica* and *Entocybe* have been reported across the world (Baroni et al. 2011; Baroni and Lamoureux 2013; da Silva et al. 2013, 2014; Park et al. 2017; Ramírez-Cruz et al. 2020a, 2020b). In China, diverse macrofungal resources have been documented (Wu et al. 2019). However, there is limited research on *Deconica* and *Entocybe*. During the present study, specimens were collected from the National Nature Reserve of Mount Tianmu located in Zhejiang Province, China from July to September 2022. Two new species, *Deconicaflavum* and *Entocyberoseoalbus* within Agaricales, are described and illustrated based on morphological and phylogenetic evidence.

## ﻿Materials and methods

### ﻿Morphological studies

Morphological observations encompassing the macro and microscopic structural characteristics were made from fresh and dried material. The color standards have been noted from the fresh samples as per Kornerup and Wanscher (1978). Microscopic characteristics were observed from dried material revived in 5% KOH, Congo red, and Patent Blue V 0.1%. The measurements were made on twenty counts each of the basidiospores (in side-view without hilum), basidia (without sterigmata), cheilocystidia, and pileipellis at 1000 × magnification per collection (Morozova et al. 2014). Spore length-width ratios were expressed as *Q*, and the average *Q* was expressed as *Q*_av_. Then, small parts of the lamellae were attached with double-sided adhesive tape on specimen holders, and coated with gold by an ion sputter coater (SBC-12, KYKY, China) for 40 s. Specimens were used to observe the spores by scanning electron microscopy (Phenom XL, Phenom-World, the Netherlands) in a low vacuum mode (15 kV). Additionally, spores of *Deconicaflavum* were measured without hilum on concave and convex sides at × 5200 magnification. Dried specimens were stored in polyethylene zipper bags and deposited in the Herbarium of Hangzhou Normal University (HTC).

### ﻿DNA extraction, PCR amplification, and determination of DNA sequences

DNA was extracted from dried basidiomata tissue using the Cetyltrimethylammonium Bromide method (CTAB) (Morozova et al. 2014). The ITS (internal transcribed spacer) including ITS1, 5.8S and ITS2, and ribosomal large subunit (LSU) regions were amplified using the primer pair ITS1–F/ITS4, and LROR/LR5, respectively (Vilgalys and Hester 1990; Gardes and Bruns 1993). Amplified PCR products were verified by 1.2% agarose gel electrophoresis stained with 4S Green Nucleic Acid (Sangon Co., Ltd., Shanghai, China) in 1 × TAE. The PCR products were sequenced by Sangon Biotech (Shanghai, China). Forward and reverse sequence reads were assembled and edited by Bio Edit v.7.0.9 from specimens (Hall 1999). The new sequences generated in this study were deposited in GenBank (http://www.ncbi.nlm.nih.gov/genbank/) and listed in Table 1.

**Table 1.** Sources of sequences and their GenBank accession numbers that were used in this study.

Species name	Specimen voucher	Country	ITS	LSU
*Clitocybesclerotoidea*	iNAT:187491457	USA	PP573968	–
*Deconicabayliasiana*	OTA:71563	New Zealand	OQ064952	–
*D.bayliasiana*	PDD:105444	New Zealand	KM975393	–
*D.bayliasiana*	OTA:73288	New Zealand	OQ065068	–
*D.chionophila*	CBS:658.87 (Type)	France	NR_160176	–
*D.chionophila*	FA 1743	France	OR419908	–
*D.citrispora*	PDD:87522	New Zealand	KM975431	–
*D.citrispora*	TENN:055373	Argentina	KY559334	–
*D.citrispora*	–	–	OL616138	–
*D.cokeriana*	CCB45 (TENN)	USA	KC669315	–
*D.cokeriana*	Ps482	USA	MK965913	–
*D.cokeriana*	PRM922477	USA	MK965914	–
*D.coprophila*	MHHNU 30335	–	MK214386	–
*D.coprophila*	257N1	–	OP237142	–
*D.coprophila*	MHHNU 7935	–	OP862790	–
*D.coprophila*	MHHNU 7937	–	OP862791	–
*D.coprophila*	S62	–	OR237579	–
***D.flavum***	**2381**	**China**	**OR906279**	**OR906277**
***D.flavum***	**2382**	**China**	**OR906280**	**OR906278**
*D.hartii*	CBS: 273.81 (Type)	Canada	MH861342	–
*D.horizontalis*	DA-17014	France	MZ234153	–
*D.horizontalis*	FF15120	France	MZ361342	–
*D.horizontalis*	FF16067	France	MZ363738	–
*D.horizontalis*	MEL:2321097	Australia	OL771718	–
*D.horizontalis*	MEL	Australia	OL771719	–
*D.horizontalis*	MEL	Australia	OL771720	–
*D.horizontalis*	S.D. Russell iNaturalist #1827064	USA	ON416969	–
*D.magica*	HN170821119	France	OM397446	–
*D.micropora*	FJ596921	–	MW871601	–
*D.milvispora*	PBM3781 (TENN) (holotype)	Australia	KC669314	–
*D.milvispora*	TENN F-067013 (holotype)	USA	NR_176108	–
*D.montana*	Hao & Guo & Han 131610	China	MH425255	–
*D.montana*	–	France	MH862108	–
*D.montana*	MICH:340541	USA	MT913618	–
*D.montana*	iNAT 37380190	USA	OM203503	–
*D.montana*	iNAT 37434339	USA	OM203504	–
*D.montana*	DAVFP:29764	Canada	OQ225666	–
*D.montana*	DAVFP:29781	Canada	OQ225683	–
*D.novae-zelandiae*	PDD:87768	New Zealand	KM975401	–
*D.overeemii*	DED 8328 (SFSU)	Africa	KX017212	–
*D.phyllogena*	SFC20160714-66	–	MF437002	–
*D.phyllogena*	Mushroom Observer # 282800	USA	MK607529	–
*D.phyllogena*	HFJAU-TD393	China	MN622718	–
*D.phyllogena*	ZMU197_ITS	China	MW724279	–
*D.phyllogena*	HBAU15299	–	MW862324	–
*D.pratensis*	L	Netherlands	MT622238	–
*D.protea*	BAP 596 (SFSU)	Africa	KX017213	–
*D.* sp.	TENN051714	USA	HQ728541	–
*D.* sp.	TFB12591 (TENN)	USA	KC669313	–
*D.* sp.	–	Thailand	KM270756	–
*D.* sp.	Mushroom Observer # 340420	USA	MK607606	–
*D.* sp.	TENN-F-009938	USA	MT622256	–
*D.* sp.	LXYZF1	–	MZ452395	–
*D.* sp.	OTA:73406	New Zealand	OQ065091	–
*D.* sp.	OTA:73424	New Zealand	OQ065098	–
*D.* sp.	FLAS-F-61579	USA	MH211973	–
*D.* sp.	RA712-7	USA	MK234215	–
*D.thailandensis*	XAL	Thailand	MT622245	–
*D.umbrina*	XAL	Malaysia	MT622246	–
*Entocybehaastii*	MEN 2004055/53	Netherlands	KC710086	–
*Entocybehaastii*	MEN 2006617	Netherlands	KC710089	–
*Entocybehaastii*	MEN 2011045	Netherlands	KC710101	–
*Entocybehaastii*	K(M):103926	UK	MF977946	–
*Entocybehaastii*	K(M):35980	UK	MF977961	–
*Entocybehaastii*	K(M):82407	UK	MF977962	–
*Entocybehaastii*	K(M):173454	UK	MF977974	–
*Entocybehaastii*	MEL:2379812	UK	MF977980	MF977980
*Entocybehaastii*	K(M):82407	UK	–	MF977962
*Entocybenitida*	F14054 (UBC)	Canada	AF335449	–
*Entocybenitida*	UBC herbarium F14288	Canada	AY228340	–
*Entocybenitida*	287	Italy	JF907989	–
*Entocybenitida*	MEN 8376	Netherlands	KC710076	–
*Entocybenitida*	Hausknecht 2006201	Netherlands	KC710100	–
*Entocybenitida*	MEN 200324	Netherlands	KC710122	–
*Entocybenitida*	iNAT:17857763	USA	OL602070	OL602070
*Entocybenitida*	iNAT:34316843	USA	OM522259	OM522259
*Entocybenitida*	ME Noordeloos 200326	Netherlands	–	GQ289175
*Entocybenitida*	NL-5402	USA	–	MK277955
*Entocybe* sp.	OMDL K. Canan iNaturalist # 185356854	USA	PP156155	–
***Entocyberoseoalbus***	**3461**	**China**	**PP974446**	**PP974447**
***Entocyberoseoalbus***	**3462**	**China**	**PP974445**	**PP974448**
*Entocybetrachyospora*	DAVFP:28111	Canada	JF899553	–
*Entocybetrachyospora*	den Bakker1153	Netherlands	KC710088	–
*Entocybetrachyospora*	den Bakker 1901	Netherlands	KC710121	–
*Entocybetrachyospora*	iNAT:17857961	USA	OL602069	OL602069
*Entocybetrachyospora*	OMDL K. Canan iNaturalist 103586037	USA	OR824557	OR824557
*Entocybetrachyospora*	TB5856	–	–	GU384629
*Entocybeturbida*	PRM 915266	Czech Republic	FJ824815	–
*Entocybeturbida*	16176	Italy	JF908005	–
*Entocybeturbida*	MEN200351	Netherlands	KC710060	–
*Entocybeturbida*	MQ18R373-QFB30889	Canada	MN992146	MN992146
*Entocybeturbida*	MQ18R118-QFB30634	Canada	MN992147	-
*Entocybeturbida*	MQ18R137-QFB30653	Canada	MN992148	MN992148
*Entocybeturbida*	F26446	Canada	MZ314674	–
*Entocybeturbida*	OMDL K. Canan iNaturalist # 188618716	USA	PP156263	PP156263
*Entocybeturbida*	TRTC175668	Canada	PP383792	–
*Entocybeturbida*	GLM 45919	Germany	–	AY207198
*Entocybeturbida*	ME Noordeloos 200351	Netherlands	–	GQ289201
*Entocybeturbida*	F26446	Canada	–	MZ314674
*Entocybevinaceum*	TB8870	–	–	GU384631
*Entolomaabortivum*	H. den Bakker 92	–	–	GQ289150
*Entolomaabortivum*	HMJAU 1955	China	–	JQ320131
*Entolomaalbotomentosum*	DA-20014	France	OM368079	OM368079
*Entolomaalcedicolor*	E. Arnolds 0276	Netherlands	–	GQ289152
*Entolomaalpicola*	TB6415	–	–	AF261302
*Entolomaameides*	RBG Kew K(M)128844	England	EU784199	–
*Entolomaassiduum*	KaiR1143	Cyprus	–	OL338157
*Entolomabaronii*	Gates E2292	Netherlands	KC710093	–
Entolomabelouvensevar.albertinae	CME5	Panama	MZ611628	MZ611628
Entolomabyssisedumvar.microsporum	SAAS1160	China	–	KU534231
*Entolomacaccabus*	ME Noordeloos 200324	–	–	GQ289155
*Entolomacetratum*	KaiR932	Austria	OL338132	OL338132
Entolomacf.vernum	RH17-107	USA	–	MW084700
Entolomacf.vernum	RH17-153	USA	–	MW084701
*Entolomacoeruleogracilis*	Gates E1777	Netherlands	KC710069	–
*Entolomacoeruleogracilis*	MEN 2004055	Netherlands	KC710107	–
*Entolomacontrastans*	L 0608161	Australia	–	MK277982
*Entolomacostatum*	G. Immerzeel 2000-10-10	Netherlands	–	GQ289161
*Entolomadepluens*	S.D. Russell ONT iNaturalist 129768621	USA	OP549186	OP549186
*Entolomaflavifolium*	TB6215	–	–	AF261301
*Entolomafuligineoviolaceum*	MEN 2009-071	Australia	–	MK277989
*Entolomagracilior*	MEN 2011043	Netherlands	KC710079	–
*Entolomagregarium*	SAAS1220 (Holotype)	China	–	KU534237
*Entolomagregarium*	SAAS1493	China	–	KU534238
*Entolomagregarium*	SAAS:1220 (Holotype)	China	–	NG_153851
*Entolomahaastii*	G. Gates E1777	Netherlands	–	GQ289168
*Entolomaheae*	SAAS1091 (Holotype)	China	–	KU534232
*Entolomaheae*	SAAS1016	China	–	KU534236
*Entolomaheae*	SAAS1091	China	–	NG_153850
*Entolomaincanosquamulosum*	MD2014-13	Italy	OL338320	OL338320
*Entolomanidorosum*	TB6263	–	–	AF261296
*Entolomanitidum*	TB7526	–	–	GU384626
*Entolomanubooccultatum*	KaiR687 (Holotype)	Panama	MZ611675	MZ611675
*Entolomaortonii*	KaiR1008	Germany	OL338141	–
*Entolomaortonii*	KaiR1008	Germany	–	OL338141
*Entolomaparaconferendum*	CME6 (Holotype)	Panama	MZ611629	MZ611629
*Entolomaparaconferendum*	CME7	Panama	MZ611630	MZ611630
*Entolomaplatyphylloides*	14740	Italy	JF908003	–
*Entolomapolitum*	ME Noordeloos 200325	–	–	GQ289181
*Entolomasericatum*	M.T. Tholl #1991	Luxembourggg	MW340721	MW340721
*Entolomasericatum*	ME Noordeloos 200328	Netherlands	–	GQ289189
*Entolomasilvae-frondosae*	L:DB6568 (Holotype)	Hungary	–	MH792065
*Entoloma* sp.	EH37	Canada	FJ717489	–
*Entoloma* sp.	T503	Australia	JF960759	–
*Entoloma* sp.	CT-4335	USA	KY462337	–
*Entoloma* sp.	S.D. Russell MycoMap 6944	USA	MK564545	MK564545
*Entoloma* sp.	TENN:077957	USA	PP831632	PP831632
*Entoloma* sp.	EM677	Japan	–	AB692015
*Entoloma* sp.	80812	China	–	KJ648486
*Entoloma* sp.	SAAS203	China	–	KJ658971
*Entoloma* sp.	SAAS712	China	–	KJ658973
*Entoloma* sp.	HGS-2021-8-23-6	–	–	OL336509
*Entolomasphagneti*	Bas 6.86	Netherlands	KC710061	–
*Entolomasphagneti*	OW-E2-14	Norway	KX945366	–
*Entolomaundatum*	16854	Italy	JF908007	–
*Entolomaundatum*	KUN-HKAS 115925 (WZ224)	China	MZ855875	MZ855875
*Entolomaundatum*	HAY-F-004639	USA	OR778327	OR778327
*Entolomaundatum*	HAY-F-002256	USA	PP575920	PP575920
*Entolomaundatum*	HAY-F-004798	USA	PP626490	PP626490
*Entolomavernum*	1193	USA	–	KX670983
*Entolomavezzenaense*	A. Hausknecht (ex WU 14588)	Netherlands	–	GQ289204
*Kuehneromyces* sp.	–	Australia	MK965912	–

Notes: - indicated no data in GenBank, the newly generated sequences are indicated in bold.

#### ﻿Phylogenetic analyses

The newly generated sequences in the study were evaluated for quality using BioEdit and Blast search results as per Nilsson et al. (2012). Relevant sequence data including outgroups in phylogenetic analyses were downloaded from the GenBank. DNA sequences were aligned and manually modified in MEGA 11 (Kumar et al. 2018). Phylogenetic analyses were performed with Maximum Likelihood (ML) and Bayesian Inference (BI) methods. ML phylogenetic analyses used IQ-Tree with 1,000 bootstrap replications (Minh et al. 2020). BI phylogenetic analyses were determined by Markov Chain Monte Carlo (MCMC) sampling using MrBayes v.3.2.7 (Ronquist et al. 2012). BI was performed with six independent MCMC runs, and trees were sampled every 100 generations. The analyses were stopped after 5,000,000 generations when the average standard deviation of split frequencies was below 0.01.

Phylogenetic trees of *Deconica* were constructed using ITS and LSU sequence data following recent publications (Noordeloos 2009; da Silva et al. 2013; Gurung et al. 2019; Ramírez-Cruz et al. 2020a). The dataset of *Deconica* consists of 59 sequences for the ITS region including outgroup *Kuehneromyces* sp. (Ps1608) (Ramírez-Cruz et al. 2020a). In ML phylogenetic analysis, the best-fitting model was TPM2u+F+R3 determined by ModelFinder (Kalyaanamoorthy et al. 2017). In BI phylogenetic analysis, the model GTR+I+G was the best substitution model which was calculated by MrMTgui (https://mrmtgui.software.informer.com/).

**Figure 1.**
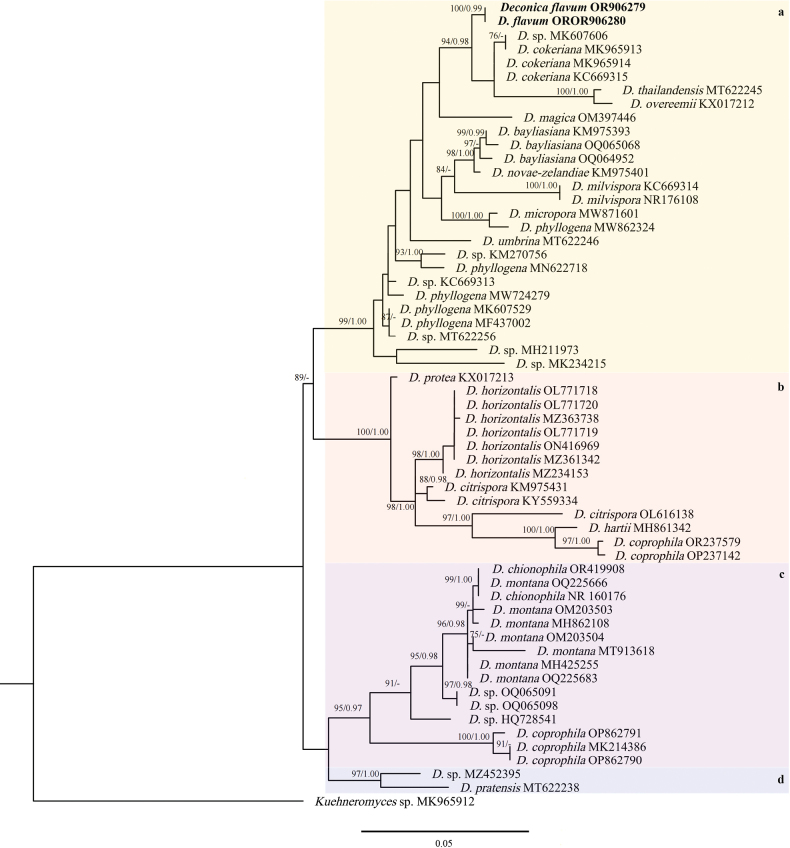
ML tree from ITS rDNA sequences of *Deconica*. Species described as new in this study are indicated in bold.

**Figure 2.**
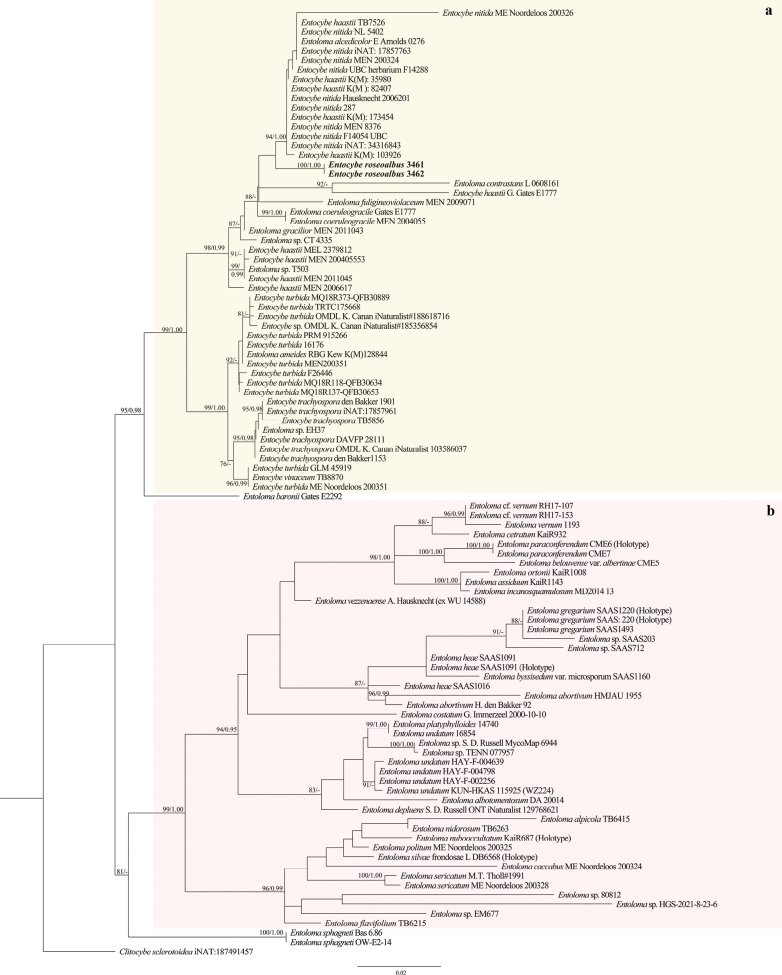
ML tree from combined ITS and LSU sequence data of *Entocybe* and *Entoloma*. Species described as new in this study are indicated in bold.

Phylogenetic trees of *Entocybe* were constructed using the two concatenated ITS–LSU sequences dataset. The dataset consists of 62 ITS sequences and 66 LSU sequences including outgroup *Clitocybesclerotoidea* (iNAT:187491457). In ML phylogenetic analysis, the best-fitting model was TIM2+F+G4 (ITS) and TIM2+F+R2 (LSU) determined by ModelFinder (Kalyaanamoorthy et al. 2017). In BI phylogenetic analysis, the best-fit model was GTR+F+G4 and K2P+I+G4 using the BIC criterion for ITS and LSU separately (Kalyaanamoorthy et al. 2017). The trees were visualized with FigTree v.1.4.0 (http://tree.bio.ed.ac.uk/software/figtree/). The tree topologies recovered by ML and BI were similar. ML bootstrap support (BS) equal to or greater than 75% and Bayesian posterior probability (PP) equal to or greater than 0.95 were shown on the nodes in Figs 1, 2.

## ﻿Results

The analyses of *Deconica* generated four supported clades, labeled a–d (Fig. 1). Clade a (BS = 99%, PP = 1.00) grouped *D.flavum*, *D.cokeriana*, *D.thailandensis*, *D.overeemii*, *D.magica*, *D.bayliasiana*, *D.novae-zelandiae*, *D.milvispora*, *D.micropora*, *D.phyllogena*, and *D.umbrina*. The clade contained six taxa with chrysocystidia i.e. *D.flavum*, *D.cokeriana*, *D.thailandensis*, *D.overeemii*, *D.milvispora*, and *D.umbrina*. Moreover, *D.flavum*, *D.cokeriana*, *D.thailandensis* and *D.overeemii* formed a subclade with strong bootstrap supports (BS = 94%, PP = 0.98) in clade a. *Deconicaflavum* differs from *D.cokeriana* (MK965913), *D.cokeriana* (MK965914), *D.cokeriana* (KC669315) and *D.* sp. (MK607606) by 7 bp, 7 bp, 8 bp and 9 bp respectively. In the clade, all the species have small basidiomata, ellipsoid, rhomboid, hexagonal, or rhomboid-nodulose basidiospores. Two collections formed an independent lineage in the trees with strong bootstrap supports (BS = 100%, PP = 0.99) in clade a (Fig. 1). Clade b (BS = 100%, PP = 1.00) included *D.protea*, *D.horizontalis*, *D.citrispora*, *D.hartii*, and *D.coprophila*. Clade c (BS = 95%, PP = 0.97) included *D.chionophila*, *D.montana*, and *D.coprophila*. In clade d (BS = 97%, PP = 1.00), *Deconica* sp. and *D.pratensis* got clustered into one branch.

The analyses of *Entocybe* resulted in two well-supported clades, clade a and clade b (Fig. 2). Clade a mainly consists of *Entocybe* species (BS = 99%, PP = 1.00). Clade b is composed of most *Entoloma* species used in this study (BS = 99%, PP = 1.00). The trees showed that the new species identified as *Entocybe* formed a well-supported clade (BS = 100%, PP = 1.00) in clade a (Baroni et al. 2011).

### ﻿Taxonomy

#### ﻿ Deconica flavum

Taxon classificationFungiAgaricalesStrophariaceae

Y.Y. Shen & Y.B. Song sp. nov.

F81F191E-EE5E-5497-B084-32785830084C

Index Fungorum: IF901538

Facesoffungi Number: FoF16635

Figs 3 , 4

##### Etymology.

The specific epithet *flavum* (Lat.) refers to the species having stramineous color in the center of the pileus.

**Figure 3.**
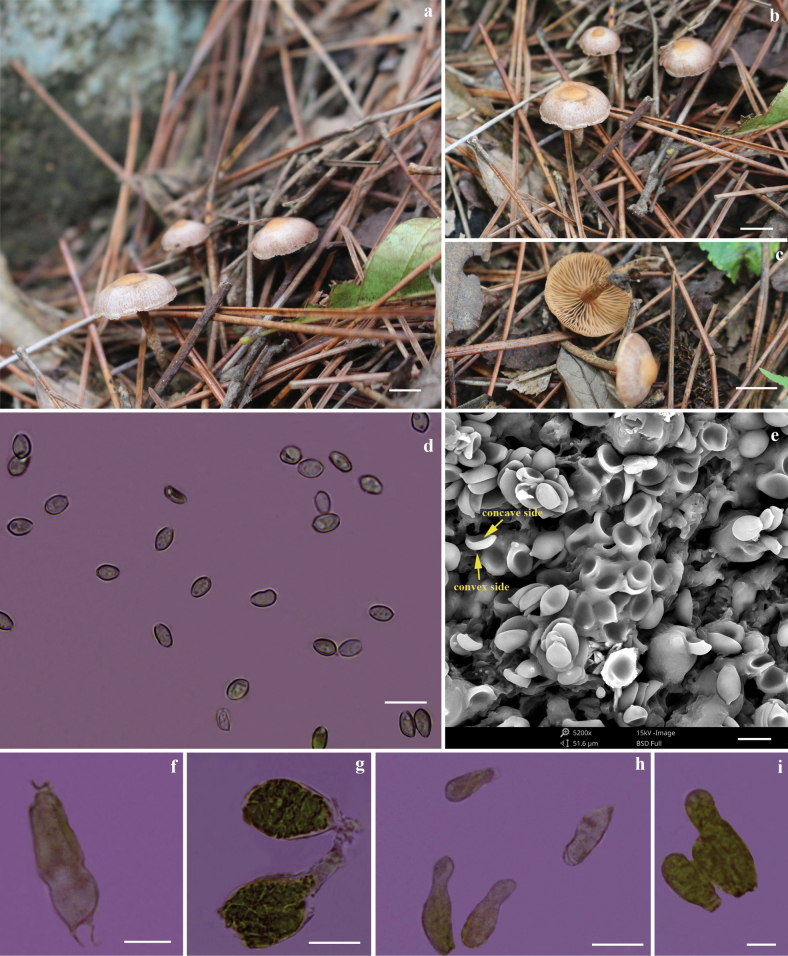
*Deconicaflavum* (holotype) **a–c** basidiomata **d–e** basidiospores **f** basidium **g** pleurocystidia type chrysocystidia **h** pleurocystidia type leptocystidium **i** cheilocystidia. Scale bars: 10 mm (**a–c**); 10 µm (**d, g, h**); 5 µm (**e, f, i**).

##### Holotype.

• China, Zhejiang Province, Hangzhou, National Nature Reserve of Mount Tianmu at 1162 m a.s.l., 30°21'N, 119°26.4'E (DDM), grew on litter under coniferous and broad-leaved mixed forest, 2 July 2022, 2381 (holotype), GenBank accessions: OR906279 (ITS), OR906277 (LSU).

**Figure 4.**
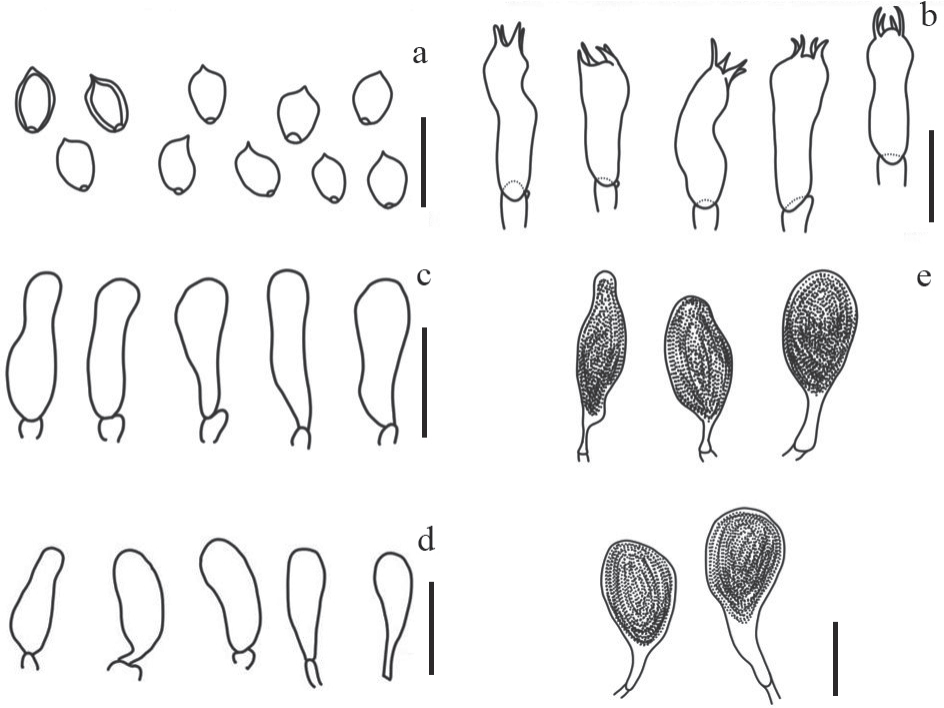
Micromorphological features of *Deconicaflavum* (holotype) **a** basidiospores **b** basidia **c** pleurocystidia type leptocystidium **d** cheilocystidia **e** pleurocystidia type chrysocystidia. Scale bars: 10 µm.

##### Description.

***Pileus*** convex-campanulate, commonly papillate, light brown (6D8) or sunburn (6D5), center stramineus (5C8), (8–)10–20 mm; margin striate, invariable color when bruised, surface flocculose or with white fibrillose patches of veil on half of the pileus. ***Context*** fleshy on disk, pale yellowish (6A3). ***Lamellae*** adnexed to adnate, with decurrent tooth, distant, pompeian yellow (5C6), brown shellow (5C8), or gold brown (5D7). ***Stipe*** central, cylindrical, equal to broader at apex, hollow, cartilaginous, flocculose, 11–14 × 1.2–2.7 mm; dark blonde (5D4) to light brown (6D5), with yellowish white fibrils. Smell indistinct.

***Basidiospores*** fusiform, ellipsoid to ovoid, yellowish brown under light microscopy with germ pore, the middle part concave under scanning electron microscopy, (3.0) 3.3–4.7 (5.1) × (2.2) 2.6–3.3 (4.3) µm, *Q* = 0.8–1.8, *Q_av_* = 1.4 (concave side), (3.6) 4.2–4.9 (5.1) × (2.9) 3.1–3.9 (4.0) µm, *Q* = 1.0–1.5, *Q_av_* = 1.3 (convex side) in frontal view. ***Basidia*** cylindrical or claviform with median constriction, 4-spored, hyaline, thin-walled, 11.7–17.1 × 3.8–5.7 µm. ***Pleurocystidia type chrysocystidia*** clavate to broadly clavate, apex mucronate or rostrate, thin-walled, hyaline, with hyaline content, 15.2–26.8 × 5.6–13.0 µm. ***Pleurocystidia type leptocystidium*** narrowly utriform, hyaline, thin-walled, abundant, 7.7–17.9 × 3.3–5.7 µm. ***Cheilocystidia*** widely utriform, cylindrical, hyaline, 12.3–20.5 × 3.6–5.2 µm. ***Pileipellis*** a gelatinous cutis 2.0–4.5 µm diam, hyaline, and thin-walled, with clamp connections. ***Stipitipellis*** a cutis 4.7–10.6 µm diam, hyaline, thin-walled, with clamp connections.

##### Habitat and distribution.

Scattered on litter under coniferous and broad-leaved mixed forests at 1162 m a.s.l., currently only known from Zhejiang Province, China.

##### Additional material examined

**(*paratype*).** • China, Zhejiang Province, Hangzhou, National Nature Reserve of Mount Tianmu at 1162 m a.s.l., 30°21'N, 119°26.4'E (DDM), grew on litter under coniferous and broad-leaved mixed forest, 2 July 2022, 2382, GenBank accessions: OR9066280 (ITS), OR906278 (LSU).

#### ﻿ Entocybe roseoalbus

Taxon classificationFungiAgaricalesEntolomataceae

Y.Y. Shen & Y.B. Song sp. nov.

3802D609-D862-51E7-A2A9-352E5F6C6C5A

Index Fungorum: IF902333

Facesoffungi Number: FoF16636

Figs 5 , 6

##### Etymology.

The specific epithet *roseoalbus* (Lat.) refers to the pinkish-white stipe.

##### Holotype.

• China, Zhejiang Province, Hangzhou, National Nature Reserve of Mount Tianmu at 1025 m a.s.l., 30°20.4'N, 119°26.4'E (DDM), grew on humus under coniferous and broad-leaved mixed forest, 2 September 2022, 3461 (holotype), GenBank accessions: PP974446 (ITS) and PP974447 (LSU).

**Figure 5.**
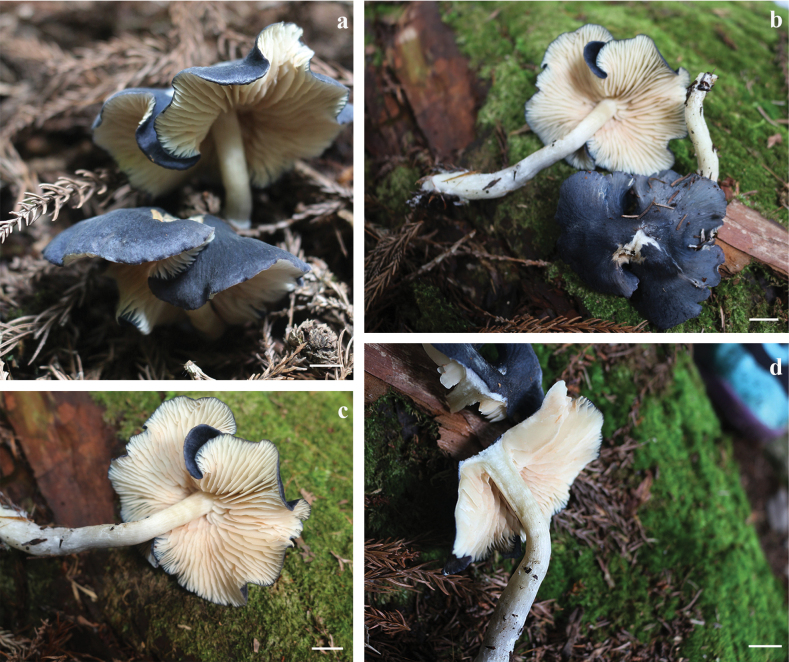
Basidiomata of *Entocyberoseoalbus* (holotype) **a** habitat of *Entocyberoseoalbus***b** whole basidiomata **c** lamellae of *Entocyberoseoalbus***d** stipe of *Entocyberoseoalbus*. Scale bars: 10 mm.

##### Description.

***Pileus*** umbonate, undulating, occasional dehiscence in the middle, and slight dehiscence at the edge when mature, not hygrophanous, not translucent-striate, surface finely felted with densely appressed-fibrillose or matted-fibrillose, rivulose, blackish blue (20F7 or 20F8) in the middle, gradually lighter, becoming dark blue (20E6), 47–68 mm diam. ***Context*** white, 1.8–1.9 mm thick above the stipe. ***Lamellae*** unequal, adnate, margin slightly serrate, 23–26 × 9.3–11.2 mm (length × breadth), at first pinkish white (7A2) then pastel red (7A4) to pale red (7A3) with basidiospore maturity. ***Stipe*** central, 69–72 mm long, 7.2–7.7 mm (apex)–7.1–7.5 mm (middle)–8.3–8.8 mm (base) diam, equal but slightly thinner in the upper middle, hollow and splits longitudinally with ease, pinkish white (10A2), white at the base, fragile. Odor not distinctive. Taste not recorded.

**Figure 6.**
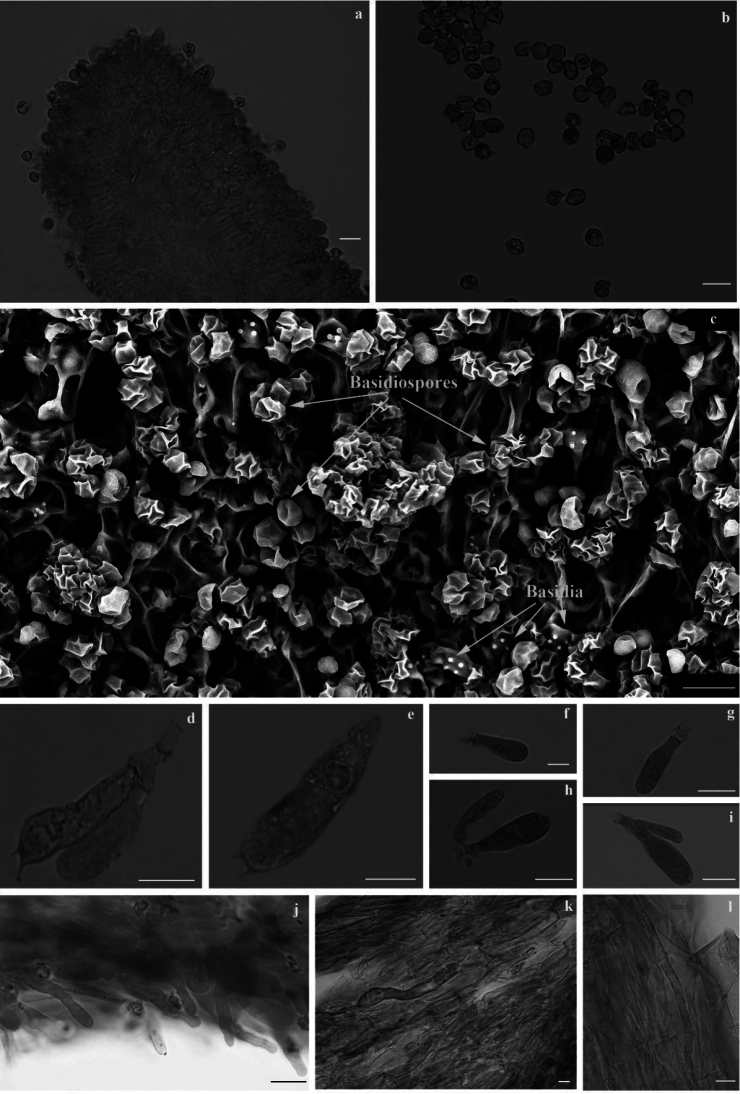
*Entocyberoseoalbus* (holotype) **a** marginal cell **b** basidiospores under the light microscope in oil (1000×) **c** basidiospores under SEM (5200×) **d–e** basidia **f–i** cheilocystidia **j** pileocystidia **k** pileipellis **l** stipitipellis. Scale bars: 10 µm.

***Basidiospores*** distinctly angular (6–8 angled) to some indistinctly and faintly rounded pustulate, ornamentation composed of broken ridges under an SEM, (4.0) 4.73–5.6 (6.5) × (4.6) 4.8–5.5 (6.1) µm, *Q* = 0.7–1.3, *Q_av_* = 1.0 in side-view. ***Basidia*** clavate, 3– or 4– sterigmate, filled with refractive oil bodies, 22.3–32.8 × 7.1–9.9 µm. Hymenial cystidia absent. ***Hymenophoral trama*** subregular, made up of cylindrical to slightly inflated elements, 36.9–92.5 × 11.6–22.8 µm. ***Lamella edge*** crowded with tufts of cheilocystidia. ***Cheilocystidia*** clavate, hyaline, abundant, 12.7–25.1 × 2.3–7.1 µm. ***Hymenial cystidia*** absent. ***Pileipellis*** multi-layered cutis, cylindrical, pigments intracellular, with special long and curved hyphae, 30.2–60.8 × 8–20.9 µm. ***Pileocystidia*** (terminal cells) narrowly cylindric to clavate, pigments intracellular, subtended by inflated cells of the pileal trama, 15.7–30.8 × 2.8–4.5 µm. ***Stipitipellis*** multi-layered cutis, similar to pileipellis, cylindrical hyphae, pigments intracellular, with special long and curved hyphae, 7.7–12.4 µm diam. ***Caulocystidia*** absent. **Clamp connections** present in all tissues.

##### Habitat and distribution.

Scatted on humus under coniferous and broad-leaved mixed forests at 1025 m a.s.l., currently only known from Zhejiang Province, China.

##### Additional material examined

**(*paratype*).** • China, Zhejiang Province, Hangzhou, National Nature Reserve of Mount Tianmu at 1025 m a.s.l., 30°20.4'N, 119°26.4'E (DDM), scatted on humus under coniferous and broad-leaved mixed forest, 2 September 2022, 3462, GenBank accessions: PP974445 (ITS) and PP974448 (LSU).

## ﻿Discussion

*Deconicaflavum* is characterized by small and convex basidiomata with ellipsoid to ovoid basidiospores, two types of pleurocystidia, chrysocystidia and leptocystidia. It was growing on litter as other *Deconica* species. *Entocyberoseoalbus* is peculiar in having isodiametric basidiospores with 6–8 angles and broken ridges, pileipellis and stipitipellis with intracellular pigment, and abundant clamp connections in all tissues, as shown by the other *Entocybe* species. Furthermore, phylogenetically in ML and BI trees, the specimens formed two distinct lineages within *Deconica* and *Entocybe*, respectively (Figs 1, 2).

*Deconica* species are distributed worldwide, with notable records in Europe, America, South Asia, and Oceania (GBIF, https://www.gbif.org/search?q=Deconica). Many species of the genus *Deconica* have been described recently based on the ITS phylogenetic analysis (Ramírez-Cruz et al. 2013, 2020a, 2020b). In the present research work, ITS and LSU sequences of *D.flavum* were generated. Due to the lack of other gene sequences (LSU), we perform only single gene (ITS) phylogenetic analysis. The phylogenetic analysis of 58 ITS sequences from *Deconica* including two newly generated sequences formed four clades with strong bootstrap supports (Fig. 1). In clade a (BS = 99%, PP = 1.00), six taxa possess chrysocystidia, four of which are clustered into a subclade with strong bootstrap supports (BS = 94%, PP = 0.98). The four species included *D.flavum*, *D.cokeriana*, *D.thailandensis* and *D.overeemii*. Although the phylogenetical distances of *D.flavum*, and *D.cokeriana* are close in ITS trees, they have distinct differences in morphological and microscopic characteristics. *Deconicaflavum* is lighter in pileus color than *D.cokeriana*, and has no discoloration when bruised. Interestingly, the center of the pileus is brow shellow, similar to “egg yolk” in *D.flavum*. Moreover, *D.cokeriana* stipe is yellowish white to light brown, with white to brownish fibrils, which become darker when bruised. While *D.flavum* is dark blonde to light brown, with yellowish-white fibrils. Microscopically, the pleurocystidia type chrysocystidia and leptocystidia of *D.cokeriana* (17–40 × 6.5–11 µm; 14–24 × 3.5–7 µm) are longer than *D.flavum* (15.2–26.8 × 5.6–13.0 µm; 7.7–17.9 × 3.3–5.7 µm). *D.thailandensis* and *D.overeemii* were originally described as *Psilocybe* species (Horak and Desjardin 2006; Horak et al. 2009). It is easy to differentiate *D.thailandensis* and *D.flavum* by basidiospores. Basidiospores of *Deconicathailandensis* and *D.overeemii*, originally described as *Psilocybe* species (Horak and Desjardin 2006; Horak et al. 2009), are rhomboid. Basidiospores of *D.flavum* are ellipsoid to ovoid, and the middle part is concave under SEM (Horak et al. 2009).

The nearly blue species in Entolomataceae, *Entocybehaastii*, *E.nitida*, *Entolomaalcedicolor*, *E.eugenei*, *E.hochstetteri*, *E.mengsongense*, *E.tadungense* and *E.virescens* have similar color in pileus and stipe (Noordeloos and Hausknecht 2007; Alves and do Nascimento 2012; Bergemann et al. 2013; Ediriweera et al. 2017). *Entocyberoseoalbus* is unique in having nearly blue pileus and yellowish grey stipe. *Entocybe* species are distributed worldwide, focused on Eastern Europe, the East and West coasts of North America, and Oceania (GBIF, https://www.gbif.org/search?q=Entocybe). The ITS–LSU phylogenetic analysis of *Entocybe* resulted in clade a and clade b with strong bootstrap support (Fig. 2). The new species, *Entocyberoseoalbus* is close to *Entocybenitida*, *E.haastii*, *Entolomaalcedicolor*, *E.contrastans*, *E.fuligineoviolaceum* and *E.coeruleogracilis* in the phylogenetical trees, which are clustered into one branch (BS = 88%, PP = –). They have distinct differences in morphological and microscopic characteristics. Compared with *Entocyberoseoalbus*, *Entocybenitida* has comparatively smaller pileus (20–40 mm), darker stipe (grayish-blue), bigger spores (7–9 × 6–8 µm), and no cheilocystidia (Noordeloos 2004). On the other hand, *Entocybehaastii* has a robust and blackish blue stipe and bigger pileocystidia (54.1–81.1 × 2.5–7.8 µm) (Bergemann et al. 2013). *Entolomaalcedicolor* has steel blue pileus and stipe and garlic odor (Noordeloos 2004). *Entolomacontrastans* has smaller mycenoid basidiomata, white pileus (8–20 mm) with a slightly darker brown center, violaceus stipe (30–50 × 2 mm), and bigger spores (6.0–8.0 × 5.5–7.5 µm) (Noordeloos 2004). *Entolomafuligineoviolaceum* has darker blue pileus and stipe, dark brown-violet to violet lamellae, and bigger spores (5.5–7.5(–8) × 5.5–6.5(–7) µm) (Noordeloos 2004). *Entolomacoeruleogracilis* has deeper blue basidiomata, smaller pileus (8–22 mm) and longer stipe (30–60 × 1–3 mm), and bigger spores (6.0–8.0 × 5.5–7.5 µm) (Noordeloos 2004).

In conclusion, sufficient evidence from morphological and molecular phylogenetic analyses supports the distinction of *D.flavum* and *Entocyberoseoalbus* from other recorded species of the respective genus.

## Supplementary Material

XML Treatment for Deconica flavum

XML Treatment for Entocybe roseoalbus

## ﻿Additional information

### Author ORCIDs

Yu-Yu Shen https://orcid.org/0009-0003-4947-4266

Zi-Wen Zhang https://orcid.org/0009-0001-4045-8505

Wen-Qian Li https://orcid.org/0009-0000-1998-030X

Xing-Ning Liu https://orcid.org/0009-0002-0176-7943

Fei-Ying Tian https://orcid.org/0009-0007-3212-5261

Chun-Mei Pang https://orcid.org/0000-0001-5346-8676

Wen-Hong Dai https://orcid.org/0009-0003-1238-3758

Yao-Bin Song https://orcid.org/0000-0001-9342-0423

Ming Dong https://orcid.org/0000-0002-1046-2140

### Data availability

Publicly available datasets were analyzed in this study which can be found here: https://www.ncbi.nlm.nih.gov/; https://indexfungorum.org/.
